# Uteroplacental Circulation in Normal Pregnancy and Preeclampsia: Functional Adaptation and Maladaptation

**DOI:** 10.3390/ijms22168622

**Published:** 2021-08-11

**Authors:** Xiangqun Hu, Lubo Zhang

**Affiliations:** Lawrence D. Longo, MD Center for Perinatal Biology, Department of Basic Sciences, School of Medicine, Loma Linda University, Loma Linda, CA 92350, USA

**Keywords:** pregnancy, preeclampsia, uteroplacental circulation, adaptation, endothelium, vascular smooth muscle

## Abstract

Uteroplacental blood flow increases as pregnancy advances. Adequate supply of nutrients and oxygen carried by uteroplacental blood flow is essential for the well-being of the mother and growth/development of the fetus. The uteroplacental hemodynamic change is accomplished primarily through uterine vascular adaptation, involving hormonal regulation of myogenic tone, vasoreactivity, release of vasoactive factors and others, in addition to the remodeling of spiral arteries. In preeclampsia, hormonal and angiogenic imbalance, proinflammatory cytokines and autoantibodies cause dysfunction of both endothelium and vascular smooth muscle cells of the uteroplacental vasculature. Consequently, the vascular dysfunction leads to increased vascular resistance and reduced blood flow in the uteroplacental circulation. In this article, the (mal)adaptation of uteroplacental vascular function in normal pregnancy and preeclampsia and underlying mechanisms are reviewed.

## 1. Introduction

Pregnancy starts from conception and ends with birth. Upon the completion of embryonic development, approximately at the end of 10 weeks of gestation, the fetus begins to grow and develop in utero [1]. The demand of nutrients and oxygen for fetal growth and development increases as pregnancy progresses, which is met by increased blood perfusion of the placenta. Depending on the species, uteroplacental blood flow at term increases 10–100-fold over nonpregnant levels [2]. To accommodate the dramatic change in uteroplacental hemodynamics, the maternal cardiovascular system undergoes physiological adaptation, as evidenced by increased plasma volume and cardiac output and decreased mean arterial blood pressure [3,4,5]. More importantly, dramatic changes occur locally. Uteroplacental circulation, which links the maternal circulation and fetal circulation, is established at the beginning of the second trimester [6,7]. The remodeling of spiral arteries and the functional adaptation of uterine arteries enable the uteroplacental circulation to become a low-resistance, high-flow system. Appropriate uteroplacental blood flow is pivotal for both fetal growth and maternal well-being [8,9]. Failure in the uteroplacental vascular transformation/adaptation is associated with pregnancy complications such as preeclampsia and fetal growth restriction [10,11,12]. Preeclampsia is characterized by new onset hypertension (systolic >140 mmHg and diastolic >90 mmHg) after 20 weeks’ gestation with one or more of the following features: proteinuria, other maternal organ dysfunction such as acute kidney injury, liver dysfunction, neurological complications and hematological complications and fetal growth restriction [13,14,15]. It affects 5–10% of pregnancies worldwide with high maternal and perinatal morbidity and mortality [12]. It also predisposes long-term health risks, especially cardiovascular and metabolic disease for the mother and child [16,17,18,19]. The remodeling of spiral arteries has been reviewed elsewhere [2,20]. Preeclampsia is a spontaneous pregnancy complication unique to humans [21]. However, due to ethical concerns and scarcity in human specimens, our understanding of the pathogenesis of preeclampsia largely relies on findings from animal models of preeclampsia induced by surgical, environmental, pharmacological, immunological or genetic manipulation before or during pregnancy which recapitulate some features of this disorder [22]. This review intends to summarize our knowledge on the functional (mal)adaptation of uteroplacental circulation in normal pregnancy and preeclampsia. To achieve this aim, relevant publications involving both human and animal model studies primarily in the past three decades were searched in PubMed and synthesized.

## 2. Uteroplacental Vascular (Mal)Adaptation in Normal Pregnancy and Preeclampsia

As uterine vascular resistance decreases, uterine blood flow increases to ~800 mL/min in late human pregnancy, from ~50 mL/min in nonpregnant subjects [23,24,25,26]. Studies in experimental animals such as sheep, guinea pigs and rats reveal that more than 80% of uterine blood flow perfuses the placenta [27,28,29]. In contrast, uterine vascular resistance is increased in preeclampsia compared to normal pregnancy, resulting in a ~50% decrease in uterine blood flow [30,31,32]. High-altitude pregnancy is associated with a ~3-fold increase in the incidence of preeclampsia [33,34]. A decrease in uterine blood flow is also observed in pregnant women at high altitude [35].

**Figure 1:**
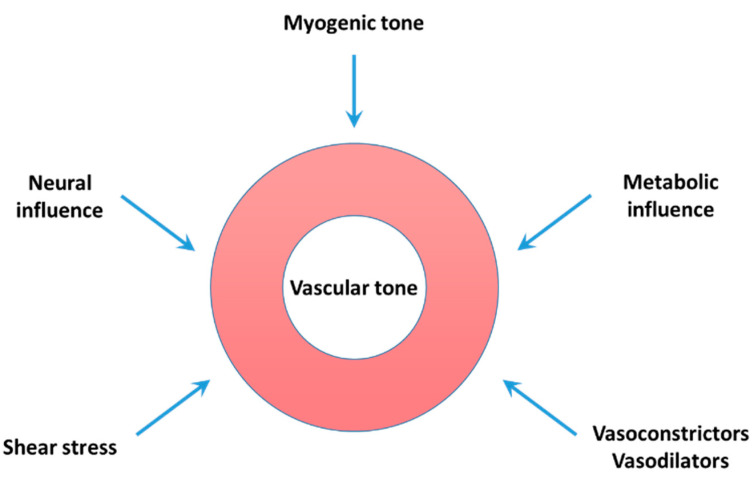
Vascular tone is determined by a variety of factors. Myogenic tone produced in response to intraluminal pressure changes constitutes the basal vascular tone, upon which vasoconstrictors, vasodilators and neurotransmitters released by sympathetic and parasympathetic nerves, locally produced metabolic substances and others can act to produce vasoconstriction or vasodilation.

In a given organ, vascular tone is largely determined by (1) intrinsic myogenic regulation; (2) the dynamics of vasoconstrictor and vasodilator influences acting on the vasculature; and (3) flow- or shear-stress-mediated regulation (Figure 1) [36]. Neurohumoral and metabolic factors also contribute to the regulation of vascular tone. Any change in these regulations could alter vascular resistance and hence blood flow to an organ. The altered uteroplacental hemodynamics in both normal pregnancy and preeclampsia is in part the consequence of functional (mal)adaptation of the uteroplacental vasculature involving both the endothelium and vascular smooth muscle.

### 2.1. Myogenic Tone

The smooth muscles of resistance arteries and arterioles possess pressure-dependent reactivity (myogenic response) [37]. They constrict upon an increase in intraluminal pressure and dilate in response to a decrease in this pressure. Myogenic tone constitutes the foundation of vascular tone and is modulated by vasodilators and vasoconstrictors, as well as other vasoactive factors. Thus, myogenic tone plays a critical role in regulating blood pressure and tissue/organ perfusion [38]. Altered uterine arterial myogenic tone may impact uteroplacental blood flow and the perfusion of the placenta. To accommodate markedly increased uterine blood flow, reduced myogenic tone of uterine arteries is desirable. Indeed, uterine arterial myogenic tone is decreased in pregnant mice [39,40]. Similarly, pregnancy-induced attenuation of myogenic tone is observed in sheep, which contributes to reduced uterine vascular tone in ovine pregnancy [41,42]. Unexpectedly, pregnancy increases uterine arterial myogenic tone in human myometrial arteries and rat radial arteries [43,44]. Intriguingly, myometrial arteries from women with preeclampsia and normal pregnancy display similar myogenic tone [45]. It should be noted that myogenic tone in myometrial arteries from preeclamptic patients is only measured at a single pressure point (80 mm Hg). Nevertheless, uterine arterial myogenic tone is increased in a rat model of preeclampsia with surgically reduced uterine perfusion pressure (RUPP) [46]. Pregnancy at high altitude is associated with increased incidence of preeclampsia [33]. Uterine arterioles from pregnant sheep at high altitude also exhibit increased myogenic tone [47,48].

### 2.2. Vasoreactivity

Pregnancy also alters the vascular reactivity of uterine arteries. Vasodilation mediated by acetylcholine and bradykinin usually requires an intact and functioning endothelium [49]. As expected, endothelium-dependent relaxation of uterine arteries in response to acetylcholine and bradykinin, as well as other compounds, is increased in pregnancy [50,51,52,53,54]. Pregnancy also enhances calcitonin gene-related peptide- and adrenomedullin-induced relaxation of uterine arteries [55,56]. On the other hand, uterine arteries from human and experimental animals become refractory to various vasopressors, including angiotensin II, endothelin, neuropeptide Y, norepinephrine, epinephrine, phenylephrine and serotonin, during pregnancy [57,58,59,60,61,62,63]. Together, these alterations may contribute to reduced uteroplacental vascular resistance in pregnancy. Apparently, the pregnancy-induced changes in uterine arterial vasoreactivity are diminished in preeclampsia. Endothelium-dependent relaxation of myometrial arteries from preeclamptic women in response to acetylcholine and bradykinin as well as other compounds is reduced [64,65,66,67,68,69,70,71,72,73]. Uterine arteries from pregnant rats exposed to chronic hypoxia also display impaired endothelium-dependent relaxation [74]. Prolonged hypoxia exposure increases uterine vascular resistance in pregnant guinea pigs [75]. In rat models of preeclampsia, induced by crossing females overexpressing human angiotensinogen with males overexpressing human renin (TgA) and by RUPP, uterine arteries exhibit both enhanced contraction to phenylephrine and repressed relaxation to acetylcholine [76,77]. Uterine arteries from TgA rats also show increased maximal contraction and sensitivity to angiotensin II [78].

### 2.3. Shear Stress

Shear stress is the frictional force on the endothelium in vasculature exerted by blood flow. Shear stress plays an important role in regulating vascular tone, angiogenesis, and vascular remodeling [79]. Shear stress is sensed by the endothelium, leading to increased release of vasodilators. In response to shear stress, the acute vascular response in peripheral circulation is vasodilation [80]. Vasodilation of uterine arteries from guinea pigs in response to shear stress increases in pregnancy [81]. NO is the major mediator of shear stress/flow-induced vasodilation of uterine arteries [45,81]. However, flow-mediated vasodilation of myometrial arteries is diminished in preeclampsia [45]. Furthermore, shear stress promotes vasoconstriction in uterine arteries of guinea pigs exposed to gestational hypoxia [81].

### 2.4. Release of Endothelium-Derived Vasodilators

Endothelial cells release various vasodilators to regulate vascular tone [82]. Notably, among them are nitric oxide (NO), prostacyclin (PGI_2_) and hydrogen sulfide (H_2_S), which are enzymatic products of endothelial NO synthase (eNOS), phospholipase A_2_ (PLA_2_)/cyclooxygenases (COXs)/prostacyclin synthase (PGIS) and cystathionine β-synthase (CBS)/cystathionine-γ-lyase (CSE), respectively. NO produces vasodilation by stimulating soluble guanylate cyclase, leading to an increase in cyclic guanosine monophosphate (cGMP) [83]. Classically, PGI_2_ exerts its vasodilation through activating PGI_2_ receptor coupled to the Gαs protein–adenylyl cyclase pathway to produce cyclic adenosine monophosphate (cAMP) [84,85]. The mechanism underlying H_2_S-induced vasodilation remains not fully understood, potentially involving activation of K^+^ channels [86]. In addition, endothelium-derived hyperpolarizing factor (EDHF) is also released by endothelial cells. EDHF plays an important role in the regulation of vascular tone mediated by the opening of Ca^2+^-activated K^+^ channels of small (SK_Ca_ or K_Ca_2.3) and intermediate (IK_Ca_ or K_Ca_3.1) conductance and myoendothelial gap junctions (MEGJs) and potassium efflux, among others [87,88]. These factors are involved in uterine vascular adaptation in normal pregnancy, whereas their abnormal production is associated with preeclampsia [89,90,91,92,93].

eNOS converts its substrate L-arginine to L-citrulline and NO, producing most vascular NO. NO plays a pivotal role in uterine vascular (mal)adaptation in pregnancy and preeclampsia [93,94]. The expression and activity of eNOS in endothelial cells of human and ovine uterine arteries are augmented in pregnancy [95,96,97]. Associated with eNOS upregulation is the increased production/release of NO in uterine arteries [95,98,99]. Uterine vascular resistance reduces following administration of the NO donor isosorbide dinitrate in pregnant women [100]. Pregnancy enhances the NOS inhibitor N^ω^-nitro-L-arginine methyl ester (L-NAME)-induced constriction of rat uterine arteries [50]. Consistently, a local infusion of L-NAME into uterine arteries reduces basal uterine blood flow in pregnant but not in nonpregnant sheep [101,102,103]. Pregnancy-mediated enhancement of endothelium-dependent relaxation and refractoriness to vasoconstrictors in uterine arteries and estrogen-induced increase in uterine blood flow are in part mediated by NO [50,54,99,104,105,106,107]. Furthermore, eNOS deficiency attenuates acetylcholine-induced endothelium-dependent relaxation of uterine arteries, increases uterine vascular resistance and reduces uterine blood flow in pregnant mice [108,109,110,111]. The expression and activity of eNOS in uteroplacental tissues in preeclampsia remain controversial and both increased [112,113] or decreased [114,115,116,117,118] expression have been documented. Chronic exposure of endothelial cells to plasma from preeclamptic patients results in increased expression/activity of eNOS [119,120]. Similarly, rat uterine arteries exposed to plasma from women with preeclampsia show an increase in eNOS expression [121]. Uterine arteries from high-altitude pregnant sheep also display upregulated eNOS [96]. However, NO bioavailability is diminished in preeclampsia [93,122]. The reduced NO bioavailability in preeclampsia is probably caused by (1) NO reacting with superoxide (O_2_^•−^) to produces peroxynitrite ONOO^−^, (2) uncoupling of eNOS, (3) substrate deficiency and (4) enzyme inhibition [114,123,124,125,126,127]. Consequently, preeclampsia diminishes shear-stress induced NO release and decreases NO-mediated dilatation in myometrial arteries [45,68]. Similarly, NO-dependent relaxation of myometrial arteries from high-altitude pregnancy is reduced [128].

CBS and CSE are two key enzymes in the synthesis of H_2_S from cysteine or homocysteine. In a way similar to NO, H_2_S is an important cardiovascular signaling molecule and contributes to the regulation of vascular tone [129]. Pregnancy promotes the expression of CBS and H_2_S production in both endothelial cells and smooth muscle cells of uterine arteries [130,131]. H_2_S-induced relaxation of human uterine arteries is increased in pregnancy [130]. E_2_β-stimulated increases in uterine blood flow, but not basal uterine flood flow, are diminished in CSE knockout mice [132]. H_2_S also relaxes placental vessels [133]. Placental expression of CBS and CSE is reduced in preeclampsia [133,134,135]. CSE expression in the placenta is also decreased in the preeclampsia model of RUPP mice [136]. Notably, siRNA knockdown of CSE in human umbilical vein endothelial cells (HUVECs) promotes the release of soluble fms-like tyrosine kinase-1 (sFlt-1) and soluble endoglin [135].

PGI_2_ is synthesized from arachidonic acid via enzymatic actions of PLA_2_, COXs and PGIS. The expression of these enzymes and production of PGI_2_ are increased in uterine arteries in pregnancy [137,138,139,140]. Infusion of PGI_2_ into uterine arteries increases uterine blood flow in pregnant dogs [141]. Although administration of the PGIS inhibitor tranylcypromine results in a reduction in uterine blood flow in pregnant sheep, the resultant uterine vascular constriction is apparently not due to reduced PGI_2_ production [142]. Placental COX1/2 expression is reduced in preeclampsia [143,144,145]. Correspondingly, PGI_2_ production is decreased, leading to a higher prostacyclin/thromboxane A2 ratio [146,147,148]. The level of 6-keto-PGF1α (a stable metabolite of prostacyclin) in omental resistance arteries is lower in preeclampsia than in normal pregnancy [149]. These findings imply a reduced role of PGI_2_ in regulating uterine vascular tone in preeclampsia.

Pregnancy enhances EDHF-mediated vasodilation of rat uterine arteries [150,151]. Myoendothelial gap junctions (MEGJs), formed by the assembly of connexins, connect endothelial cells and juxtaposed vascular smooth muscle cells to make them electrically coupled. An increase in intracellular Ca^2+^ in endothelial cells activates IK_Ca_ and SK_Ca_, leading to hyperpolarization. MEGJs enable endothelial cell hyperpolarization to be directly transmitted to vascular smooth muscle cells to cause subsequent vasorelaxation [152]. EDHF through MEGJs appears to be the major mediator of endothelium-dependent relaxation of myometrial arteries in pregnancy, which is impaired in preeclampsia [71,153,154]. SK_Ca_ channel expression and SK_Ca_ channel-mediated relaxation of uterine arteries are suppressed in high-altitude pregnant sheep [155]. The expression of IK_Ca_ and SK_Ca_ channels in placental chorionic plate arteries is downregulated in preeclampsia, leading to impaired IK_Ca_- and SK_Ca_-mediated vasorelaxation [117]. The expression of SK_Ca_ channels in HUVECs is also reduced in preeclampsia [156]. Exposing human uterine microvascular endothelial cells and HUVECs to plasma from preeclamptic patients also causes downregulation of the SK_Ca_ channel [156]. This downregulation is mediated by NOX2-derived superoxide.

## 3. Mechanisms Underlying Uterine Vascular (Mal)Adaptation in Normal Pregnancy and Preeclampsia

### 3.1. The Estrogen-ER Signaling Pathway

Circulating estrogen level increases progressively and substantially during pregnancy [157]. Estrogen is a key player in regulating uteroplacental vascular function, leading to reduced uterine vascular resistance/increased uterine blood flow in pregnancy [158,159,160,161,162]. Direct evidence of regulation of uterine blood flow by estrogen comes from observations that infusion of the non-selective estrogen receptor inhibitor ICI 182, 780 into the uterine artery reduces basal uterine blood flow in pregnant sheep and that both acute and chronic administration of estradiol-17β (E_2_β) increase uterine blood flow in ovariectomized nonpregnant sheep [163,164,165]. The reduction in exogenous and endogenous estrogen-induced increase in uterine blood flow by ICI 182, 780 suggests the involvement of estrogen receptors (ERs) [165]. Not surprisingly, activation of either ERα or ERβ relaxes both human myometrial and placental arteries [166]. G-protein-coupled estrogen receptor (GPER, also known as G-protein-coupled receptor 30 (GPR30)) is expressed in rat uterine arteries and its expression is increased in pregnancy [167]. Consistently, pregnancy enhances the relaxation of rat uterine arteries mediated by GPER [167]. However, this mechanism seems not to occur in human uteroplacental vessels, as the selective GPER agonist G1 fails to relax both myometrial and placental arteries [166]. Estrogen also induces ER-independent relaxation of rat uterine arteries [168]. The estrogen effects on the endothelium and vascular smooth muscle of uteroplacental vessels are evidently mediated by impacting multiple signaling pathways, as discussed below.

The placenta is the primary source of estrogen during pregnancy [169]. Preeclamptic patients have lower placental and plasma estrogen compared to the counterparts of normal pregnancy [169,170,171,172]. Similarly, high-altitude pregnancy also displays lower circulating estrogen [173,174]. Deficiency of aromatase in the preeclamptic placentas has been shown to account for the reduced biosynthesis of estrogen [175,176]. Hypoxia apparently mediates the downregulation of aromatase in the placenta [176,177]. In addition, elevated ROS in preeclamptic placentas also suppresses estrogen biosynthesis [172]. The aberrant estrogen production in turn disrupts the E_2_β-ER signaling pathway and plays an important role in the pathogenesis of preeclampsia [161,169].

Estrogen exerts its regulatory actions by binding to multiple ERs, including classical nuclear ERα and ERβ as well as membrane GPER [178]. Estrogen generally stimulates its target genes, including its own expression by (1) ligand-activated ER binding to the estrogen response element (ERE) in the target gene, and (2) ligand-activated ER tethering with the other transcription factors. ER expression in uterine arteries is regulated by estrogen status. Both ERα and ERβ are expressed in human and ovine uterine arteries and their expression is increased in pregnancy [166,179,180]. Their upregulation in pregnancy is stimulated by estrogen, as it is replicated by E_2_β administration in ovariectomized nonpregnant sheep and rats and by ex vivo E_2_β treatment of uterine arteries from nonpregnant ewes [179,180,181]. A half ERE consensus-binding site is located in the ESR1 promoter [182] and its role in regulating ER expression in uterine arteries remains unexplored. It appears that the second mechanism is responsible for the upregulation of ERα in ovine uterine arteries in pregnancy. A study from the Zhang lab demonstrates that both ERα and ERβ could tether with Sp1 at the Sp1^−520^-binding site in the promoter of the Erα-encoding gene ESR1 to regulate ERα expression in ovine uterine arteries [183]. The Sp1^−520^-binding site is hypermethylated in the nonpregnant status, preventing Erα-SP1 binding to the Sp1 binding site. Pregnancy promotes the demethylation of the site, leading to increased ERα expression in uterine arteries, which is probably due to the estrogen-mediated upregulation of ten-eleven translocation methylcytosine dioxygenase 1 (TET1), an enzyme catalyzing active demethylation [184]. The expression of ESR1 is reduced in preeclamptic placentas, whereas the placental expression of Erβ-encoding gene ESR2 is upregulated in preeclampsia [185,186]. The downregulation of ESR1 is induced by exposing human placenta-derived BeWo cells to hypoxia [185]. Similarly, the expression of ESR1 in ovine uterine arteries is also reduced in high-altitude pregnancy as the result of hypoxia [181,183]. Hypoxia upregulates DNA methyltransferase 3b (DNMT3b) and downregulates TET1, leading to ESR1 promoter hypermethylation and subsequent downregulation of ESR1 in uterine arteries of pregnant sheep [187,188,189]. E_2_β stimulates GPER expression in HTR8/SVneo cells [190]. The expression of GPER is also reduced in preeclamptic placenta [190].

**Figure 2:**
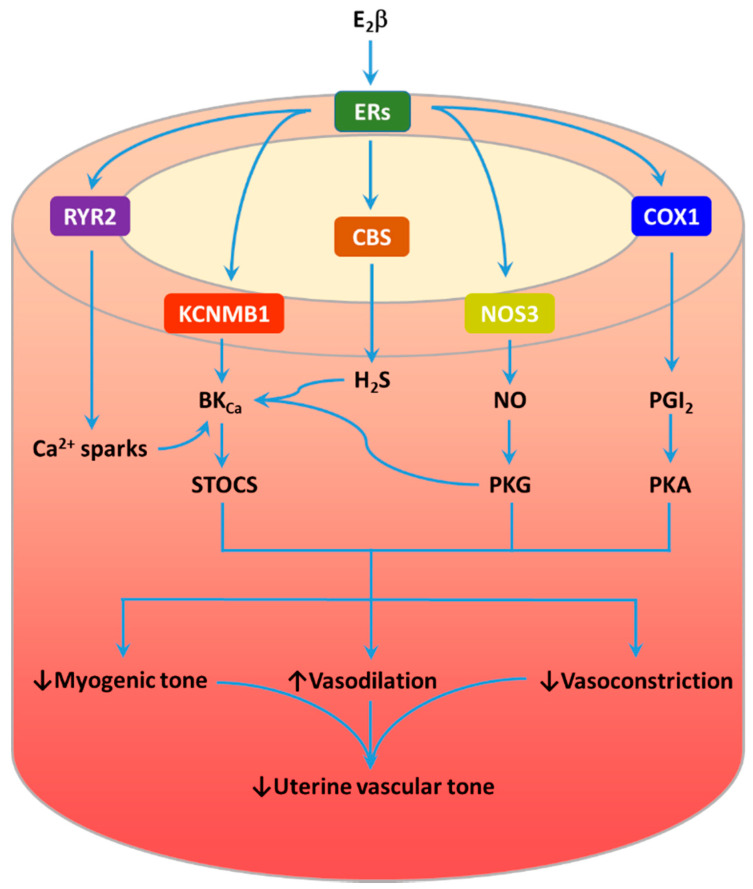
Estrogen plays a pivotal role in regulating functional adaptation of uteroplacental vessels in pregnancy. 17β-Estradiol (E_2_β) upregulates RYR2 and KCNMB1 expression and ryanodine receptor 2 (RyR_2_) and large-conductance Ca^2+^-activated K^+^ (BK_Ca_) channel activity and subsequently enhances Ca^2+^ spark/STOC coupling, leading to reduced uterine arterial myogenic tone. E_2_β also increases production/release of nitric oxide (NO), hydrogen sulfide (H_2_S) and prostacyclin (PGI_2_) via upregulating the expression of NOS3, CBS and COX1 in uterine arteries. These vasodilators, through receptor or nonreceptor mechanisms to activate protein kinase A (PKA) and protein kinase G (PKG), promote vasodilation and blunt vasoconstriction, resulting in an overall decrease in uterine vascular tone.

eNOS is a downstream signal of the estrogen-ER signaling pathway. Acute estrogen exposure stimulates NO production/release from endothelial cells of ovine uterine arteries by regulating stimulatory and inhibitory phosphorylation sites of eNOS [191]. Activation of ERα increases phosphorylation in eNOS^Ser1177^ and eNOS^Ser635^ and decreases phosphorylation in eNOS^Thr495^, whereas activation of ERβ only reduces phosphorylation in eNOS^Thr495^. Chronic treatment with E_2_β in ovariectomized nonpregnant sheep increases eNOS expression in the endothelium of uterine arteries through transcriptional regulation [192,193,194]. In HUVEC, E_2_β-induced upregulation of eNOS is mediated by ERα [195] (Figure 2). The upregulation of CBS is evidently mediated by the E_2_β–ER signaling pathway (Figure 2). Administrating E_2_β into ovariectomized nonpregnant sheep promotes CBS expression in both endothelial cells and vascular smooth muscle cells of uterine arteries [196]. Moreover, the upregulation of CBS by E_2_β is blocked by ICI 182, 780 in cultured uterine artery endothelial cells and smooth muscle cells [197,198]. Exposure of villus explants to hypoxia–reoxygenation upregulates miR-21, which in turn downregulates CSE [133]. E_2_β is also found to stimulate COX-1 and PGIS through ERα in HUVECs and ovine uterine artery endothelial cells, leading to increased PGI_2_ production [199,200] (Figure 2). EDHF-mediated vasodilation of uterine arteries is also subject to E_2_β modulation. E_2_β replacement in ovariectomized nonpregnant rats increases EDHF-mediated vasodilation of uterine arteries via increasing Ca^2+^ signal in endothelial cells [201]. Consistently, the expression of SK_Ca_ channels in ovine uterine arteries is upregulated by E_2_β [155]. Pregnancy also through the E_2_β-ERβ pathway upregulates the expression of angiotensin II type 2 (AT_2_) receptor in the endothelium of human and rat uterine arteries, leading to increased uterine blood flow in rats [202,203]. Evidently, the dysfunction of estrogen biosynthesis can have great impacts on the expression/activity of eNOS, CBS/CSE, COXs/PGI, SK_Ca_ channels and AT_2_ receptors in uteroplacental vessels. Estrogen also regulates other signal pathways, which is discussed below.

### 3.2. Ca^2+^ Spark/STOC Coupling

Myogenic tone in resistance arteries/arterioles is regulated by both global and local Ca^2+^ in vascular smooth muscle cells [38]. An increase in intraluminal pressure results in membrane depolarization and opening of the L-type Ca^2+^ channel Ca_V_1.2. The subsequent increase in global Ca^2+^ concentration due to Ca^2+^ influx activates contractile proteins in vascular smooth muscle and increases contractions. On the other hand, increased intraluminal pressure also leads to localized and concentrated Ca^2+^ release events (Ca^2+^ sparks) mediated by ryanodine receptors (RyRs) in the sarcoplasmic reticulum of vascular smooth muscle cells. Ca^2+^ sparks in turn activate adjacent BK_Ca_ channels in the plasma membrane, producing spontaneous transient outward currents (STOCs). The β1 subunit of the BK_Ca_ channel functions as the sensor of Ca^2+^ sparks and transmits the Ca^2+^ signal to the BK_Ca_ channel [204]. K^+^ efflux carried by STOCs causes membrane hyperpolarization and closure of Ca_V_1.2, resulting in vasorelaxation. Thus, the Ca^2+^ spark/STOC coupling is an important mechanism to regulate myogenic tone and blood flow [205,206]. Pregnancy has been shown to lower ovine uterine arterial myogenic tone by promoting Ca^2+^ spark/STOC coupling [207,208]. This is achieved by estrogen-mediated upregulation of the expression/activity of BK_Ca_ channel β1 subunit and RyR2 [42,207,209,210] (Figure 2). As expected, basal uterine blood flow in nonpregnant sheep is negligibly affected by BK_Ca_ channel inhibitor tetraethylammonium [102]. However, basal uterine blood flow in pregnant sheep and estrogen-induced increase in uterine blood flow in both nonpregnant and pregnant ewes are dramatically reduced by the BK_Ca_ channel blockade [102,211,212]. Furthermore, pregnancy-induced decrease in uterine vascular resistance is absent in mice with genetically deleted BK_Ca_ channel α subunit [213].

Both BK_Ca_ channel α and β1 subunits are expressed in human and ovine uterine arteries [42,102,214]. Only the β1 subunit, but not the α subunit, in uterine artery is upregulated in ovine pregnancy [42,210]. The expression of the γ1 subunit is similarly increased in mouse uterine artery [213]. The γ1 subunit also increases BK_Ca_ channel activation and promotes vasodilation of uterine arteries. Notably, the BK_Ca_ channel mediates uterine vasodilation induced by NO, H_2_S, calcitonin gene-related peptide and adrenomedullin [55,56,210,214,215]. However, the vasodilation of human placenta vessels induced by H_2_S is mediated by NO and ATP-sensitive K^+^ (K_ATP_) channels [133]. Interestingly, the BK_Ca_ channel can be activated by E_2_β through directly binding to the β1 subunit [216,217]. Hence, the increased uterine blood flow in response to E_2_β probably in part is mediated by directly activating the BK_Ca_ channel in uterine arteries. The BK_Ca_ channel also contributes to the refractoriness to vasoconstrictors in uterine arteries in pregnancy. Phenylephrine-induced contraction of ovine uterine arteries is potentiated by the BK_Ca_ channel inhibitor tetraethylammonium [210]. Similarly, phenylephrine infusion-induced increase in uterine vascular resistance in pregnant sheep is enhanced by tetraethylammonium [218]. Pregnancy increases AT_2_ receptor expression in the endothelium of uterine arteries in pregnant rats, which is associated with blunted uterine vasoconstriction to angiotensin II [202]. As BK_Ca_ channel activity is enhanced by activating AT_2_ receptor [219], the refractoriness of uterine arteries to angiotensin II in pregnancy is probably mediated by AT_2_ receptor-stimulated BK_Ca_ channel activity. Moreover, PKC-mediated vasoconstriction of ovine uterine arteries is enhanced by inhibiting the BK_Ca_ channel with tetraethylammonium [220]. Overall, the activation of the BK_Ca_ channel functions as a negative feedback mechanism to limit excessive vasoconstriction. However, the β1 subunit is downregulated in human placental chorionic plate arteries and in HUVECs from preeclamptic patients [116,221]. High-altitude pregnancy also suppresses β1 subunit expression in ovine uterine arteries [48]. The downregulation of the β1 subunit in preeclampsia could contribute to the increased uteroplacental vascular resistance and reduced uteroplacental blood flow.

The expression of BK_Ca_ channel β1 subunit-encoding gene KCNMB1 in ovine uterine arteries is determined by the dynamics of DNA methylation and demethylation. In uterine arteries of nonpregnant sheep, the Sp1-binding site (Sp1^−380^) at the KCNMB1 promoter is hypermethylated, which blocks transcription factor binding and inhibits KCNMB1 expression [222]. The CpG methylation at Sp1^−380^ is reduced in pregnancy owing to E_2_β–ER signaling-mediated upregulation of TET1 expression/activity [184]. The demethylation allows ERα and Sp1 co-binding, leading to enhanced KCNMB1 expression and hence channel activity. However, Ca^2+^ spark/STOC coupling in uterine arteries is suppressed in high-altitude pregnancy due to hypoxia-mediated suppression of E_2_β-induced upregulation of KCNMB1 and RYR and the direct effect of hypoxia on both genes via increased DNA methylation and/or miR-210-mediated degradation of KCNMB1 and RYR2 [48,222,223].

### 3.3. HIFs, Oxidative Stress and Endoplasmic Reticulum Stress

Uteroplacental tissues exhibit a hypoxic phenotype in preeclampsia as evidenced by the similarity in global gene expression in placentas from preeclamptic patients and high-altitude pregnancy and in placentas exposed to hypoxia in vitro [224]. Expression of hypoxia inducible factors (HIFs) is increased in preeclamptic placentas and in uterine arteries from high-altitude pregnant sheep [225,226]. DNMT3b contains a HIF-1α binding site in its promoter [227]. The upregulation of DNMT3b in uterine arteries of high-altitude pregnant sheep is probably mediated by HIF-1, leading to hypermethylation of ESR1 and KCNMB1 and suppressed expression [181,187]. In addition, miR-210 is a direct target of HIFs and is upregulated in both preeclamptic placenta and ovine uterine arteries from high-altitude pregnancy [188,228,229]. High-altitude pregnancy also induces oxidative stress and endoplasmic reticulum stress in human placentas and ovine uterine arteries [223,230,231,232]. Likewise, chronic hypoxia induces endoplasmic reticulum stress in rat placentas [233]. These altercations probably work concertedly, leading to the downregulation of BK_Ca_ channel β1 subunit and RyR2 expression/activity and the subsequent increase in uteroplacental vascular tone. For example, hypoxia through HIF-1 triggers ESR1 and KCNMB1 promoter hypermethylation by inducing DNMT expression and by reducing TET1 expression via miR-210-mediated mRNA degradation/translation inhibition [181,188,189], thus suppressing ESR1 and KCNMB1 expression in ovine uterine arteries in high-altitude pregnancy. In addition, miR-210 also directly targets KCNMB1 and RYR2, causing their degradation [234]. Moreover, ROS could directly suppress BK_Ca_ channel activity in ovine uterine arteries from high-altitude pregnancy [226,232]. Furthermore, endoplasmic reticulum stress has been shown to decrease the protein abundance of BK_Ca_ channel β1 subunit by promoting ubiquitin ligase-mediated degradation of the β1 subunit in vascular smooth muscle cells [235]. Intriguingly, whereas both oxidative stress and endoplasmic reticulum stress suppress Ca^2+^ spark/STOC coupling, only oxidative stress disrupts estrogen-mediated regulation of STOCs in ovine uterine arteries from high-altitude pregnancy [234].

### 3.4. Kinase Signaling

Protein kinases are important regulators of vascular contractility through phosphorylation of target proteins [236,237]. In general, activation of PKG induces vasorelaxation, whereas activation of protein kinase C (PKC) promotes vasoconstriction. Uterine vascular function is also subject to modulation by protein kinases.

It is well established that NO induces vasorelaxation by stimulating soluble guanylyl cyclases to generate cGMP, which in turn activates PKG [238]. Activation of PKG has been shown to augment Ca^2+^ spark/STOC coupling by increasing Ca^2+^ sparks and/or increased BK_Ca_ channel activity through phosphorylation, resulting in reduced myogenic tone [239,240,241,242]. BK_Ca_ channel activity is stimulated by PKG in uterine arterial vascular smooth muscle cells [102]. Along with increased eNOS expression and NO production, cGMP, PKG and BK_Ca_ channel activity are all increased in the uterine arteries of pregnant sheep [210]. Expectedly, the NO donor sodium nitroprusside increases STOCs in uterine arterial vascular smooth muscle cells from pregnant sheep (unpublished data). In addition, activation of PKG also blunts uterine vasoconstriction [243]. The expression of PKG is reduced in decidua form preeclamptic patients [244]. The downregulation of PKG is probably induced by chronic hypoxia [245]. High-altitude pregnancy also impairs PKG-mediated modulation of the BK_Ca_ channel by reducing the association of PKG with BK_Ca_ channels in vascular smooth muscle cells of ovine cerebral arteries [246].

PKC is an important mediator of vasoconstriction induced by various vasoconstrictors [237,247]. PKC contributes to vascular contractility through regulating ion channels and ultimately [Ca^2+^]_i_, increasing Ca^2+^ sensitivity of the contractile proteins and activating Ca^2+^-independent contraction [237]. In guinea pig uterine arteries, PKC is a major contributor to vasocontraction induced by norepinephrine [248] and probably to endothelin-1 and angiotensin II, as seen in the other vascular beds [247]. Activation of PKC has been shown to inhibit Ca^2+^ spark frequency in cerebral arteries [249] and to suppress BK_Ca_ channel activity in uterine arteries [42]. PKC activity and its signal pathway in ovine and rat uterine arteries and other vessels are reduced in pregnancy, apparently due to E_2_β’s action [41,250,251,252,253]. As expected, the downregulation of PKC activity contributes to reduced uterine arterial myogenic tone in ovine pregnancy [41]. However, the E_2_β-mediated downregulation of PKC activity in ovine uterine arteries is diminished in high-altitude pregnancy owing to hypoxia-induced suppression of E_2_β-ER signaling, resulting in increased PKC activity [180,254]. Similarly, HUVECs exposed to serum from preeclamptic patients display elevated PKC activity [255]. The elevated PKC activity in turn inhibits BK_Ca_ activity [220]. Consequently, vasoconstriction to PKC activation and myogenic tone in uterine arteries are increased in uterine arteries from high-altitude pregnancy [180,256].

### 3.5. Angiogenic Balance

Vascular endothelial growth factor (VEGF) and placental growth factor (PlGF), members of the VEGF family, are predominantly expressed in the placenta. Their expression in the placenta increases as pregnancy progresses [257]. Both of them play a vital role in angiogenesis [258,259]. In addition, they are also potent vasodilators and participate in regulating uterine vascular tone [257,260,261]. Local overexpression of VEGF increases uterine blood flow in pregnant sheep and reduces uterine vasoconstriction to phenylephrine, which is accompanied by increased levels of phosphorylated eNOS^Ser1177^ [262,263,264]. Similarly, VEGF also increases phosphorylation of eNOS^Ser1177^ in HUVECs [265]. These observations suggest that VEGF initiates vasodilation via stimulating NO release. Indeed, the vasodilation of rat uterine arteries induced by VEGF and PlGF is primarily mediated by NO [257,261]. Pregnancy through the E_2_β-ER signaling pathway enhances VEGF-induced vasodilation of rat uterine arteries [257,266]. VEFG-stimulated eNOS activity and production of NO and H_2_S are enhanced in human and ovine pregnancy [267,268,269]. A 24 h incubation of human uterine arteries with PlGF also blunts angiotensin II-induced vasoconstriction [270]. sFlt-1 also belongs to the VEGF family and is a splice variant of the VEGF receptor Flt1 lacking the cytoplasmic and transmembrane domains. In preeclamptic patients, levels of sFlt-1 in both the placenta and blood are increased [271,272,273,274,275]. The increased expression of sFlt-1 in the preeclamptic placenta is mediated by HIFs [276,277,278]. sFlt-1 functions as a scavenger of VEGF and PlGF and reduces the bioavailability of VEGF and PlGF [279,280], despite that circulating VEGF is increased owing to hypoxia in preeclampsia [281,282,283]. As expected, the circulating level of PlGF is reduced in preeclampsia [271,274]. Elevated sFlt-1 in the circulation leads to endothelial dysfunction [280]. Not surprisingly, exposure of bovine aortic endothelial cells to sFlt-1 and serum from preeclamptic patients inhibits mitochondrial respiration and increases mitochondrial ROS production [284]. In addition, VEGF-stimulated phosphorylation of eNOS^Ser1177^ in HUVECs is reduced by sFlt-1 [265]. Moreover, prolonged treatment of human uterine arteries with sFlt-1 enhances vasoconstriction to angiotensin II [270]. The role of sFlt-1 in the pathogenesis of preeclampsia is corroborated by the finding that chronic infusion of sFlt-1 into pregnant rats produces a preeclampsia phenotype [285].

### 3.6. Inflammation

Tumor necrosis factor α (TNFα) is a potent mediator of inflammatory and immune functions. In preeclampsia, the production/release of TNFα in the placenta is increased [286,287,288]. Correspondingly, circulating TNFα level increases in women with preeclampsia [289,290,291,292]. Hypoxia or hypoxia/reoxygenation is found to stimulate TNFα production in human placental villous explants [293,294]. Uteroplacental vascular function is impaired by the increased circulating TNFα. TNFα promotes mitochondrial ROS production and eNOS downregulation in HUVECs [295,296,297]. Pregnancy enhances sustained Ca^2+^ bursts and eNOS activity in ovine uterine artery endothelial cells by promoting connexin 43 function, which is suppressed by TNFα [298,299]. Further evidence supporting the involvement of TNFα in uterine vascular dysfunction in preeclampsia comes from animal studies. Chronic administration of TNFα promotes preeclamptic symptoms such as hypertension and proteinuria in pregnant baboons and rats [300,301]. Pregnant mice exposed to chronic intermittent hypoxia display uterine vascular dysfunction as evidenced by reduced endothelium-dependent vasodilatation and enhanced vasoconstriction, which are associated with increased plasma TNFα and sFlt-1 [302]. In two rat models of preeclampsia, administration of the TNFα inhibitor etanercept restores uterine vascular function by increasing endothelium-dependent vasorelaxation to acetylcholine and decreasing vasoconstriction to norepinephrine in uterine arteries of RUPP rats and stroke-prone spontaneously hypertensive rats, resulting in reduced uterine vascular resistance [303,304].

### 3.7. Autoimmunity

Angiotensin II type 1-receptor autoantibody (AT_1_-AA) is an agonistic autoantibody to the AT_1_ receptor. AT_1_-AA induces vasoconstriction via activating AT_1_ receptor [305]. Preeclamptic patients also display elevated AT_1_-AA in the circulation [274,305,306,307]. The detection of AT_1_-AA in serum is associated with abnormal uteroplacental perfusion [308]. Chronic infusion of AT_1_-AA in pregnant rats increases uterine vascular resistance [309]. Notably, the elevated uterine vascular resistance is decreased by ‘n7AAc’, a capped inhibitory peptide binding to the AT_1_-AA and blocking AT_1_-AAs from binding to the AT_1_ receptor [310]. Prolonged incubation of HUVECs with serum from preeclamptic patients suppresses mitochondrial respiration and increases mitochondrial ROS [311]. The increased mitochondrial ROS following exposure to serum from preeclamptic patients is reduced by ‘n7AAC’ [311]. Similarly, the mitochondrial ROS in HUVECs induced by serum of RUPP rats is also lowered by ‘n7AAC’ [312]. AT_1_-AA inhibition in RUPP rats by administration of ‘n7AAc’ reduces mitochondrial ROS in the placenta and ablates preeclamptic symptoms [310,312]. In a preeclampsia model induced by immunizing pregnant BALB/c mice with AT_1_-AA, the expression of the BK_Ca_ channel β1 subunit is reduced in mesenteric arteries [313].

## 4. Conclusions

Uteroplacental vessels undergo adaptation to accommodate increased uteroplacental blood flow in pregnancy. Observations from human and animal studies suggest that this adaptation involves functional alterations in both vascular smooth muscle cells and endothelial cells. E_2_β appears to be the primary mediator of the functional adaptation in pregnancy, increasing the Ca^2+^ spark/STOC coupling, eNOS expression/activity and cGMP–PKG signaling pathway, to name a few. These changes ultimately lead to reduced uterine vascular resistance. However, the functional adaptation of uterine arteries is impaired by the aberrant E_2_β production and its associated signaling pathways and by bioactive factors produced in preeclampsia. Extensive research, including clinical and experimental studies, has enriched our understanding of the functional changes of uteroplacental vasculature in physiological and pathophysiological conditions of pregnancy. However, our knowledge is still incomplete, which requires us to explore further. It also remains challenging to translate laboratory discoveries into patient care for preeclampsia, and more efforts are needed to close the gap between the bench and the bedside.
